# Infrared Fluorescent Imaging as a Potent Tool for *In Vitro*, *Ex Vivo* and *In Vivo* Models of Visceral Leishmaniasis

**DOI:** 10.1371/journal.pntd.0003666

**Published:** 2015-03-31

**Authors:** Estefanía Calvo-Álvarez, Kostantinos Stamatakis, Carmen Punzón, Raquel Álvarez-Velilla, Ana Tejería, José Miguel Escudero-Martínez, Yolanda Pérez-Pertejo, Manuel Fresno, Rafael Balaña-Fouce, Rosa M. Reguera

**Affiliations:** 1 Departamento de Ciencias Biomédicas, Universidad de León, Campus de Vegazana, León, Spain; 2 Centro de Biología Molecular “Severo Ochoa”, Universidad Autónoma de Madrid, Madrid, Spain; 3 Diomune, Parque Cientifico de Madrid, Madrid, Spain; United States Food and Drug Administration, UNITED STATES

## Abstract

**Background:**

Visceral leishmaniasis (VL) is hypoendemic in the Mediterranean region, where it is caused by the protozoan *Leishmania infantum*. An effective vaccine for humans is not yet available and the severe side-effects of the drugs in clinical use, linked to the parenteral administration route of most of them, are significant concerns of the current leishmanicidal medicines. New drugs are desperately needed to treat VL and phenotype-based High Throughput Screenings (HTS) appear to be suitable to achieve this goal in the coming years.

**Methodology/Principal findings:**

We generated two infrared fluorescent *L*. *infantum* strains, which stably overexpress the *IFP 1*.*4* and *iRFP* reporter genes and performed comparative studies of their biophotonic properties at both promastigote and amastigote stages. To improve the fluorescence emission of the selected reporter in intracellular amastigotes, we engineered distinct constructs by introducing regulatory sequences of differentially-expressed genes (*A2*, *AMASTIN* and *HSP70 II*). The final strain that carries the *iRFP* gene under the control of the *L*. *infantum HSP70 II* downstream region (DSR), was employed to perform a phenotypic screening of a collection of small molecules by using *ex vivo* splenocytes from infrared-infected BALB/c mice. In order to further investigate the usefulness of this infrared strain, we monitored an *in vivo* infection by imaging BALB/c mice in a time-course study of 20 weeks.

**Conclusions/Significance:**

The near-infrared fluorescent *L*. *infantum* strain represents an important step forward in bioimaging research of VL, providing a robust model of phenotypic screening suitable for HTS of small molecule collections in the mammalian parasite stage. Additionally, HSP70 II+*L*. *infantum* strain permitted for the first time to monitor an *in vivo* infection of VL. This finding accelerates the possibility of testing new drugs in preclinical in *vivo* studies, thus supporting the urgent and challenging drug discovery program against this parasitic disease.

## Author Summary

Visceral leishmaniasis (VL), caused by *Leishmania infantum* or *L*. *donovani*, is still one of the most threatening diseases affecting poor people in developing countries, with a fatality rate as high as 100% in two years in infected and untreated people. With no vaccine available and ineffective and toxic chemotherapy, the search for new potential drugs that accelerate the urgent drug discovery process are highly needed. A novel technology that addresses this important issue has been developed, by performing High Throughput Screening (HTS) assays in 384-well plates format in combination with an infrared *L*. *infantum*-expressing strain. The system was further validated to identify active compounds against VL in an *ex vivo* splenic culture. In addition, *in vivo* non-invasive imaging of the visceral infection in BALB/c mice was achieved for the first time by using transgenic fluorescent parasites. These findings open up the possibility of testing vast amounts of potential compounds and allow *in vivo* screening of drug candidates against this severe parasitic disease in an attempt to speed up the vital drug discovery program.

## Introduction

Neglected Tropical Diseases (NTDs) are severe scourges that affect the less protected layer of the poorest population of low-income countries [1, 2]. Their neglected consideration implies a poor attention by most of the actors involved in their eradication including Pharma Industries, which are more concerned about those diseases affecting people from more developed countries [3]. The fight against these diseases is mainly based on preventive measures, but when the latter fails and a sudden outbreak emerges, no effective vaccines exist and treatment is based on the administration of drugs [4]. However, due to the low investments in R & D to develop new compounds most of them are outdated, toxic in many cases, full of undesirable side effects and their route of administration requires hospitalization [5, 6]. All these are major drawbacks for the frequently overwhelmed health systems of these countries.

Recent drug discovery programs sponsored by public or private initiatives pursue a crushing defeat of major NTDs during this decade. Visceral leishmaniasis (VL) is one of the diseases that accomplish all the conditions to be a NTD. Unfortunately, no effective vaccine candidates, either prophylactic or preventive, are under clinical trials [7], the treatment is still mainly based on old-fashioned antimony derivatives and the administration route of these drugs is parenteral. To overcome these gaps, several Big Pharma companies have made available to academic researchers and supranational institutions myriads of small molecules to be tested on *Leishmania* on recently developed High Throughput target-based and target-free Screenings platforms (HTS) [8, 9]. High Content Screening (HCS) image-based readouts using confocal microscopy, or genetically modified parasites expressing easily detectable reporters are in the pipeline of target-free (phenotypic) *in vitro* screenings [10, 11]. The use of transgenic light-emitting parasites has an additional advantage since they permit a further scaling up to *in vivo* preclinical trials using rodent models of both visceral and cutaneous infections for monitoring parasite loads by means of bioimaging devices [12].

So far, luminescent transgenic parasites that express genes encoding the firefly or *Renilla* sp. luciferases are the only systems that permit a rapid readout *in vitro* under HTS conditions and the assessment of parasitic burdens in internal organs of living mice [13–15]. The major pitfall of luminescence is the need of adding a light-emitting substrate–luciferin or coelenterazine–that is time-consuming and significantly increases the cost of the analysis. For this reason, standard fluorescent proteins are more suitable for *in vitro* assays [16]. There are many drug screening systems using GFP, RFP or mCherry-transformed parasites that have been useful to evaluate libraries of compounds on *Leishmania* promastigotes and amastigotes [17–19]. Moreover, the appraisal of *in vivo* infections by using these genetically-modified strains is currently limited to cutaneous leishmaniasis (CL) models. However, fluorescence emission in the visible spectrum has low tissue penetration to be recorded by the standard optical imaging platforms [20]. This drawback has been overcome by the arising of a new developed protein (IFP 1.4) from the bacteriophytochrome of *Deinococcus radiodurans*, whose emission in the near-infrared region prevents any background interference derived from organs or tissues [21]. This protein has been successfully used to image the *in vivo* infection of the adenovirus serotype 5 (Ad5) that specifically infects the mouse liver *in vivo*. More recently, a new-engineered infrared fluorescent protein (iRFP) from the photosynthetic bacterium *Rhodopseudomonas palustris* showed a clear superiority over IFP 1.4 to evidence the viral load of Ad5 in mouse liver [22].

In this report, we have established for the first time a platform based on the comparative analysis of IFP 1.4- and iRFP- transfected *L*. *infantum* BCN 150 amastigote-infecting splenocytes *ex vivo* to perform phenotypic screenings with several drug collections of small molecules. In addition, the latter strain was used to monitor the development of an *in vivo* infection of VL, showing the higher tissue penetration of iRFP over IFP 1.4, which represents a useful tool to assess the parasite load by non-intrusive bioimaging techniques.

## Methods

The animal research described in this manuscript complies with Spanish Act (RD 53/2013) and European Union Legislation (2010/63/UE). The used protocols were approved by the Animal Care Committee of the University of León (Spain), project license number (PI12/00104).

### Mice and parasites

Female BALB/c mice (6–8 weeks old) were obtained from Harlan Interfauna Iberica SA (Barcelona, Spain) and housed in specific-pathogen-free facilities for this study. L. infantum (strain MCAN/ES/96/BCN 150) promastigotes were obtained from J.M. Requena (Centro de Biología Molecular Severo Ochoa, Madrid, Spain). Parasites were routinely cultured at 26ºC in M199 medium supplemented with 25 mM HEPES pH 6.9, 10 mM glutamine, 7.6 mM hemin, 0.1 mM adenosine, 0.01 mM folic acid, 1x RPMI 1640 vitamin mix (Sigma), 10% (v/v) heat-inactivated foetal calf serum (FCS) and antibiotic cocktail (50 U/ml penicillin, 50 μg/ml streptomycin).

### Generation of near-infrared *L*. *infantum* strains

The 987-bp IFP1.4 coding region was amplified by PCR from pENTR1A vector, a kindly gift from Dr. Roger Y. Tsien, Departments of Pharmacology and Chemistry & Biochemistry, UCSD (USA). The oligonucleotides used as primers (RBF696 and RBF687 in Table 1) introduced NcoI-NotI as restriction sites. The 948-bp iRFP coding region was digested with BglII and NotI from pShuttle CMV-iRFP vector obtained from Dr. Vladishlav V. Verkhusha −Department of Anatomy and Structural Biology and Gruss-Lipper Biophotonics Center, Albert Einstein College of Medicine, Bronx, New York (USA). The successful cloning of these ORFs in pLEXSY-hyg2 (Jena Bioscience) yielded pLEXSY-IFP1.4-HYG and pLEXSY-iRFP-HYG vectors, respectively. Parasites expressing IFP1.4 and iRFP reporters were obtained after electroporation of *L*. *infantum* BCN150 promastigotes with the linear SwaI-targeting fragment obtained from the above described vectors. Subsequent plating on semisolid media containing 200 μg/ml hygromycin B, allowed the isolation of individual clones that were subcultured in liquid media with antibiotic pressure. Correct integration of each fragment into the 18S rRNA locus of the resulting clones (IFP 1.4+*L*. *infantum and* iRFP+*L*. *infantum*) was confirmed by PCR amplification analysis, using appropriate primers (Table 1).

**Table 1:** Oligonucleotides used in this work.

Oligo No.	Sequence^a^ ^,^ ^b^	Purposed^c^	
RBF696	ccgctcgagCCATGG **CCACC**ATGGCTCGGGACCCTCTGCC	IFP 1.4	F
RBF697	ataagaatGCGGCCGCTCATTTATACAGCTCGTCCATTCC	IFP 1.4	R
RBF843	ATGGCGGAAGGATCCGTCGC	iRFP	F
RBF844	TCACTCTTCCATCACGCCGATC	iRFP	R
RBF674	ataagaatGCGGCCGC ATCGCCCGAGTGCTGTGAAAC	3’ HSP70	F
RBF675	gcTCTAGAAGATCTTGACGGGTGAATGTGTTT	3’ HSP70	R
RBF774	gcTCTAGA **CCACC**ATGACCGAGTACAAGCCCACG	PAC	F
RBF775	ggACTAGTTCAGGCACCGGGCTTGCGG	PAC	R
RBF847	ataagaatGCGGCCGCGGCTCGGCGTCCGCTTTCC	3’A2	F
RBF848	ggACTAGTGGTTATGCTTATGTGGGCGTAC	3’A2	R
RBF861	ataagaatGCGGCCGCGTGGCTGTCTAACTACACTTTCG	3’Amastin	F
RBF862	ggACTAGTGCGGCTCGCCAGTGTAGCAG	3’Amastin	R

^a^Underlined sequence indicates restriction site.

^b^Bold sequence indicates optimized translation initiation sequence.

^c^Orientation of primers: F, forward; R, reverse.

To achieve a higher stability of the reporter mRNAs 3’-untranslated regions (3’-UTRs) and intergenic regions (IR) were also included. For this purpose, three fragments derived from *L*. *donovani* A2 (LinJ.22.0670) (3400 bp) [23], *L*. *infantum* AMASTIN (LinJ.34.1010) (1200 bp) [24] and HSP70-II (LinJ.28.3060) (1600 bp) [25], were amplified using forward primers, which introduced a 5’-*NotI* site, and reverse primers that introduced a 3’-*XbaI* site (Table 1). Each amplicon was *NotI/XbaI*-digested and ligated into the parental construct pLEXSY-iRFP-HYG previously cut with the same enzymes to remove the urt2, thus yielding pLEXSY-iRFP-A2-HYG, pLEXSY-iRFP-AMASTIN-HYG and pLEXSY-iRFP-HSP70-HYG, respectively. Electroporation and semisolid selection, as well as correct integration, were done as described above. Several clones from each electroporation were grown in liquid medium in the presence of antibiotic selection and those clones with higher infrared fluorescent emission were selected after flow cytometry analysis (Cyan ADP, Dako). In addition, each *L*. *infantum* modified strain was used to infect mice in order to recover the lost infectivity after cloning and plating. We will hereafter refer to these strains as A2+*L*.*infantum*, AMASTIN+*L*.*infantum* and HSP70+*L*. *infantum*.

### *In vitro* infections

The human acute leukemia monocyte cell line (THP-1, ATCC TIB-202), was cultivated in RPMI medium supplemented with 10% heat-inactivated FCS and 1% streptomycin/penicillin at 37ºC and 5% CO_2_. The cultures were diluted every 3 or 4 days to maintain the cell density between 10^5^ cells/ml and 8 x 10^5^ cells/ml. THP-1 cells at 5 x 10^5^ cells/ml were differentiated with 50 ng/ml of phorbol 12-myristate 13-acetate (PMA) for 48 h at 37ºC and 5% CO_2_. Stationary promastigotes of each infrared strain (5–6 day cultures) freshly transformed from lesion amastigotes were added to differentiated-macrophages at 10:1 ratio for 24 h at 37ºC. Then, the parasites that have not been internalized were removed by washing with phosphate-buffered saline (PBS). Intracellular infrared signal was analyzed after 72 h post-infection by flow cytometry (Cyan ADP, Dako).

### *In vivo* infections, spleen cell proliferation and parasite quantification

Female BALB/c mice (5 animals per group) were injected intraperitoneally with either 10^7^ infective-stage metacyclic promastigotes of *L*. *infantum* BCN 150 wild type, IFP 1.4+, iRFP+, A2+, AMASTIN+ and HSP70+*L*. *infantum* strains. Metacyclic parasites were purified from stationary cells freshly transformed from lesion amastigotes by negative selection with peanut agglutinin [26]. At different times post-infection, five animals were euthanized and their spleens and livers were aseptically recovered, washed in cold PBS and placed in Petri dishes. Spleens were used for evaluating the proliferative response of spleen cells after concanavalin A (Con A) addition. Briefly, small pieces were obtaining by using a scalpel. In order to obtain a single cell suspension, the tissue was incubated with 2 ml of collagenase D (Roche) at 2 mg/ml of buffer (10 mM HEPES pH 7.4, 150 mMNaCl, 5 MmKCl, 1 mM MgCl_2_, 1.8 mM CaCl_2_) for 20 min at 37ºC. The cell suspension and remaining tissue fragments were gently passed through a 100 μm-cell strainer to remove the tissue fragments. After erythrocyte lysis, splenocytes were washed 3x with PBS by centrifugation (500 x g for 7 min at 4ºC). Splenocytes were seeded at a density of 2 x 10^5^ cells/well in 96-wells plates in RPMI medium, 20% FCS, 1 mM sodium pyruvate, 1x RPMI vitamins, 10 mM HEPES and 100 U/ml penicillin and 100 μg/ml streptomycin supplemented with 50 μM 2-mercaptoethanol. Cells were cultured in medium alone (control) or stimulated with Con A (5 mg/ml) for 3 days. One μCi of [^3^H]-thymidine was added for the final 16 to 18 h of the culture. Subsequently, cells were washed, lysed and [^3^H]-thymidine incorporation was measured onto glass fiber filters for scintillation counting. The splenocyte stimulation index was determined by dividing the cpm of Con A-stimulated and non-stimulated splenocytes [27].

The total number of living parasites in the organs was calculated from single-cell suspensions that were obtained by homogenization of the tissue through a wire mesh. Briefly, liver and spleen homogenates (100 mg/ml) were serially diluted in complete Schneider’s medium and distributed in 96-well plates at 26ºC. After 10 days, each well was examined and categorized as positive or negative according to the presence of viable promastigotes. The number of parasites was calculated as follows: Limit Dilution Assay Units (LDAU) = (geometric mean of titer from quadruplicate cultures) x (reciprocal fraction of the homogenized organ added to the first well). The titer was the reciprocal of the last dilution in which parasites were observed [28].

### Extraction of intracellular parasites from *in vitro* and *ex vivo* infections and their further processing

Intracellular amastigotes were purified from infected THP-1 macrophages and from spleen tissues in order to evaluate the infrared emission of intracellular parasites and for developing a standard curve up to the fifth week post-infection (5 wpi) in mice. On the one hand, 72 h post-infection THP-1 macrophages were washed twice with cold PBS, and scraped off using a rubber policeman. On the other hand, the spleens at different time points were collected and a splenocyte suspension was obtained by passing the spleen through a wire mesh. Both cellular suspensions from *in vitro* infections and from splenocytes were disrupted by passing sequentially through 27G^1/2^ and 30G^1/2^ needles, and polycarbonate membrane filters having pore sizes of 8 μm, 5 μm and 3 μm (Isopore, Millipore) [29]. Released amastigotes (free of host cells), were washed twice with PBS (3000 x g for 10 min at 4ºC) and counted by direct microscopy. The standard curves were developed using microscopic quantification of amastigotes and the corresponding infrared signal at 2-fold serial dilutions.

### Development of the *ex vivo* splenic explant culture

Several parameters were included in the development of the explant culture. i) Parasite infection rate is not equal in all animals from the same batch, thus in order to normalize the *ex vivo* culture, only spleens with weights ≥ 0.7 g were selected to prepare the *ex vivo* explants. ii) The starting cell density was adjusted in terms of fluorescence emission in a wide range of arbitrary units (0.25, 0.5, 1.0, and 2.0 x 10^5^ AU). Since we intend to evaluate the drug inhibition in *ex vivo* cultures for at least 4 days, the initial number of AU that matched this condition was chosen. iii) Discrimination between drug-treated and untreated control cells was assessed by adding amphotericin B (AmpB) to the explants cultures. iv) To prevent the edge effect caused by evaporation from outer wells in assays with multi-day incubations, the top and bottom rows as well as the first and last columns were filled with ddH_2_O. In addition, the positive and negative controls were symmetrically distributed through the columns [30].

### Screening of compound collections

A total of 298 compounds belonged to three distinct collections including i) indenoisoquinolines obtained from Dr. Mark S. Cushman, Department of Medicinal Chemistry and Molecular Pharmacology (Purdue University, Indiana, USA.); ii) podophylotoxin and quinones were a kindly gift from Dr. Arturo San Feliciano, Deparment of Química Farmacéutica (Universidad de Salamanca, Spain) and iii) carbolins obtained from Dr. Sankaran Murugeshan, Department of Pharmacy, (Birla Institute of Technology & Science, Pilani, India). Stock solutions consisted of test compound at a concentration of 200 μM in 20% DMSO. The 200 μM master plates were further diluted by transferring aliquots of 5 μl into black 384-well plates with clear bottom containing 45 μl of culture medium. The final concentration of DMSO in both assay and control wells not exceeded 1% (v/v). Splenocytes obtained from infected mice (50 μl), were added to Testing Plates (containing the compounds to be test at single 10 μM concentration) and Control Plates (containing positive and negative controls). The plates were incubated at 37ºC and 5% CO_2_ and at 0, 24, 48 and 72 h the infrared signal was recorded using the Odyssey infrared system (LI-COR, USA). The anti-leishmanial reference drugs were amphotericin B, miltefosine, and paromomycin sulfate.

The antileishmanial effect of the compounds was calculated as the percentage inhibition in relation to AmpB and DMSO as follows: (mean of 1%DMSO–Experimental Value) / (mean of 1%DMSO–mean of AmpB_EC100_) x 100. The positive control (10 μM AmpB), and the negative control (1% DMSO), placed in columns 2 and 23, were included in all Testing Plates. To normalize compound activity in relation to plate-to-plate variations in the assay signal, and to calculate Z’-factors, Control Plates containing also 10 μM AmpB and the vehicle 1% DMSO (in alternating fashion) were also included [30]. The quality of the assay was given by Z’ factor that was calculated as 1 –[(3SD positive controls + 3SD negative controls) / (mean of the positive controls–mean of the negative controls)]. Data from plates were used only if Z’ factors were > 0.5 [31]. Primary hits were defined as compounds displaying 60% inhibition of signal readout.

### Assessment of cell toxicity (CC_50_)

To test the toxicity of the identified hit compounds we established splenic *ex vivo* explant cultures of uninfected BALB/c mice. Briefly, the animals were euthanized and the spleens were aseptically removed and homogenized as described above. The cell suspension culture was conducted in RPMI medium, 10% FCS, 1 mM sodium pyruvate, 1x RPMI vitamins, 10 mM HEPES and 100 U/ml penicillin and 100 μg/ml streptomycin. Cells were microscopically counted and distributed into clear 96-well plates; 50 μl of uninfected splenocytes were added to each well (5 x 10^5^ cells), containing 50 μl of serial 2-fold dilutions of the test compounds (200–0.1 μM) or the DMSO control. After 72 h of incubation at 37ºC and 5% CO_2_, the viability of the cells was assessed using the Alamar Blue assay according to manufacturer’s recommendations (Invitrogen).

### *In vivo* fluorescence imaging of infrared *L*. *infantum* infected BALB/c mice

Mice infected with wild type and HSP70+*L*. *infantum* parasites were imaged using an intensified charged coupled device camera of the In Vivo Imaging System (IVIS 100, Xenogen). To minimize background when imaging in the near infrared, the normal diet was replaced by a purified diet during 7 days (AIN-93M, LabDiet, UK), since chlorophyll fluoresces naturally, emitting between 675 and 685 nm, and is detected in the 700 nm channel. Nevertheless, animals were fasted overnight, although water was allowed freely before acquiring the images in order to assure that faeces were removed from intestines. The animals were shaved to reduce background signal due to the fur. The animals were then lightly anesthetized with 2.5–3.5% isoflurane (then reduced to 1.5–2.0%), placed in the camera chamber, and the fluorescence signal was acquired for 1 s. Fluorescence determinations, recorded by the IVIS 100 system, were expressed as a pseudocolour on a grey background, with yellow colour denoting the highest intensity and dark red the lowest one.

## Results

### Generation and characterization of infrared-*L*. *infantum* strains

Aimed to create stably-integrated infrared *L*. *infantum* BCN 150 strains we electroporated wild-type promastigotes with the lineal 6156 and 6110 bp SwaI-SwaI fragments containing the ORFs encoding *IFP 1*.*4* and *iRFP*, respectively, as well as the selection marker of the pLEXSY-hyg2 plasmid. We will refer to them as IFP 1.4+*L*. *infantum* and iRFP+*L*. *infantum*. After selection on semisolid plates containing 200 μg/ml hygromycin B, individual colonies were seeded in M199 liquid medium supplemented with 10% FCS and hygromycin B. Genomic DNA isolated from these cultures was used to confirm the correct integration of the target sequence into the 18S rRNA locus of *L*. *infantum* genome (data not shown).

**Fig 1:**
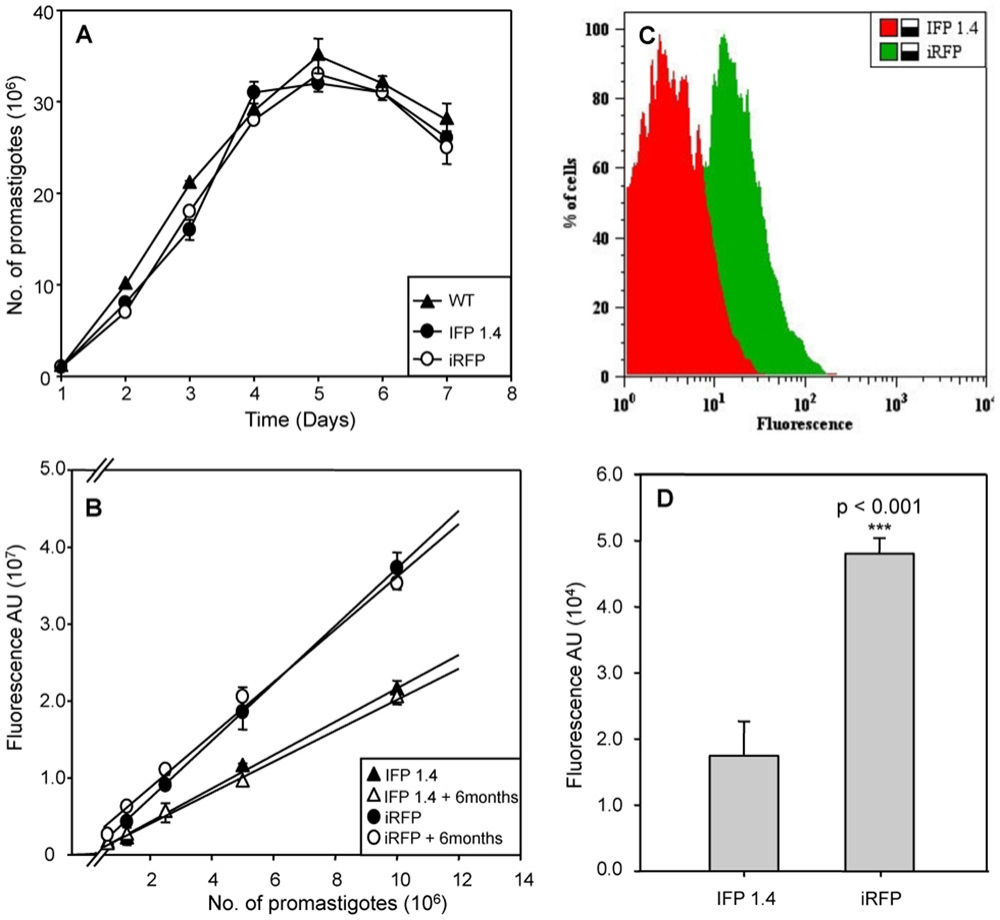
Infrared *IFP1*.*4* and *iRFP* genes are functionally expressed in *L*. *infantum* parasites. A) Growth rate of wild-type (WT; ▲) and stably-modified promastigotes IFP1.4+*L*.*infantum* (●) and iRFP+*L*.*infantum* (○). Parasites were counted using a Coulter counter. B) Correlation between fluorescence signal expressed as arbitrary units (AU) and the number of logarithmic IFP1.4+*L*. *infantum* (●) and iRFP+L. *infantum* (○) promastigotes in the presence of hygromycin B or after 6 months culture without drug IFP1.4+*L*. *infantum* (▲), iRFP+L. *infantum* (Δ). Two-fold serial dilutions were applied. C) Flow-cytometry analysis of intracellular amastigotes isolated from THP-1 *in vitro* infections with IFP1.4+*L*. *infantum* (red) and iRFP+*L*. *infantum* (green) strains. D) Mean fluorescence intensity emitted by lesion-derived amastigotes obtained from the engineered parasites. Experiments were carried out with 10^7^ parasites by triplicate and error bars represent standard deviations. Significance level *** p< 0,001; ** p<0.01; from two tails of Student *t*-test.

Fig. 1A shows that the genomic manipulation of the engineered strains did not affect the growth rate of the parasites compared to wild-type promastigotes. Further comparative studies aimed to characterize the two infrared strains were performed. In order to measure the fluorescence emission of both strains, we employed logarithmic promastigotes of IFP 1.4+*L*. *infantum* and iRFP+*L*. *infantum* (Fig. 1B), as well as intracellular amastigotes isolated from infected THP-1 cells *in vitro* (Fig. 1C), and lesion-derived amastigotes obtained from infected spleens of BALB/c mice at 5 wpi (Fig. 1D). A clear positive correlation was observed between the fluorescence signal and the number of logarithmic promastigotes placed in 96-well plates (Fig. 1B). Surprisingly, intracellular parasites expressing both infrared reporters (1C and 1D) displayed higher differences than promastigote cultures. All results clearly showed the superior fluorescence signal of the iRFP protein over IFP1.4, particularly at the really relevant stage of the parasite life cycle, the amastigotes, both isolated from THP-1 macrophages and from lesions. Moreover, the stability of iRFP expression was monitored over a period of 6 months after transfection and no change was observed in fluorescence intensity during this period, even in the absence of hygromycin B (Fig. 1B).

### Construct optimization

**Fig 2:**
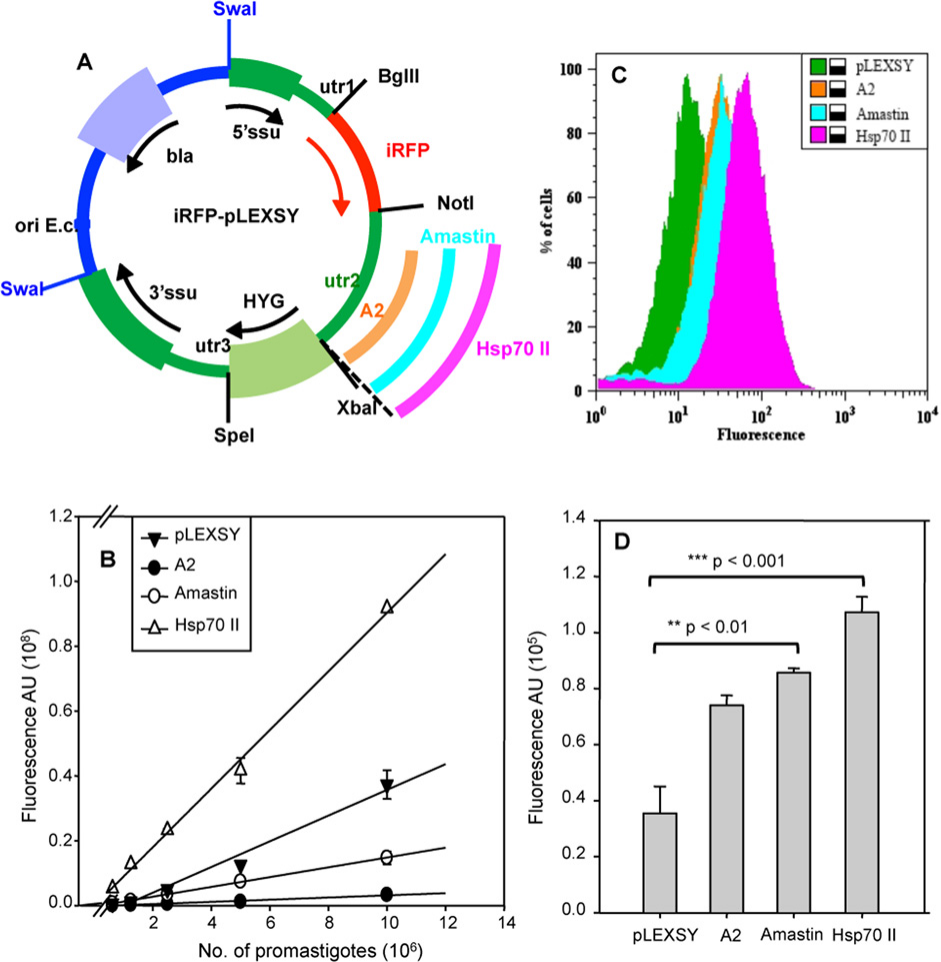
Optimization of pLEXSY-iRFP vector by introducing downstream regions (DSR) of different genes. A) Schematic view of pLEXSY-hyg2 constructs containing infrared (iRFP) reporter gene and different DSR corresponding to *A2*, *AMASTIN* and *HSP70-II* genes. Constructs were linearized by *SwaI* digestion prior to electroporation. B) Correlation between fluorescence signal and the number of logarithmic promastigotes modified with iRFP-pLEXSY-HYG (□) or carrying the DSR of *A2* (●), *AMASTIN* (○) or *HSP70-II* (Δ) genes. C) Flow-cytometry analysis of intracellular amastigotes isolated from THP-1 *in vitro* infections. D) Mean fluorescence intensity emitted by lesion-derived amastigotes obtained from WT and the engineered parasite strains carrying iRFP. Experiments were carried out with 10^7^ parasites by triplicate and error bars represent standard deviations (SD). Significance level *** p< 0,001; ** p<0.01; from two tails of Student *t*-test.

Once the iRFP reporter was chosen, we optimized the expression by replacing the original utr2 from pLEXSY-hyg2 vector with three sequences containing both 3’UTRs and IR (we will refer to as downstream regions, DSR). Two of these DSR belong to stage-regulated genes in the amastigote stage, the *A2* and *AMASTIN* genes [23, 24]. The third DSR belongs to the *HSP70 II* gene of *L*. *infantum*, a sequence involved in mRNA stability at 37ºC [25]. Fig. 2A shows the schematic view of the different constructs containing the iRFP reporter gene.

In a similar way to IFP 1.4 and iRFP-expressing parasites, we proceeded to compare the fluorescent signal of iRFP+, A2+, AMASTIN+ and HSP70+*L*. *infantum* strains. A clear correlation between the fluorescence emission and the number of parasites was also detected by using logarithmic promastigotes (Fig. 2B). Interestingly, replacement of the utr2 from pLEXSY vector with the corresponding DSR sequences from the *A2* and *AMASTIN* genes resulted in a reduction of the fluorescent levels in the promastigotes. However, using the same strains but isolating the intracellular amastigote stage, from both infected THP-1 cultures (Fig. 2C) and lesions (Fig. 2D), we observed a 2–2.5-fold increase in fluorescence emission. Nevertheless, the most significant increase (p<0.001) was obtained with amastigotes carrying the plasmid including HSP70 II as DSR.

### Characterization of a VL murine model with HSP70+*L*. *infantum*

**Fig 3:**
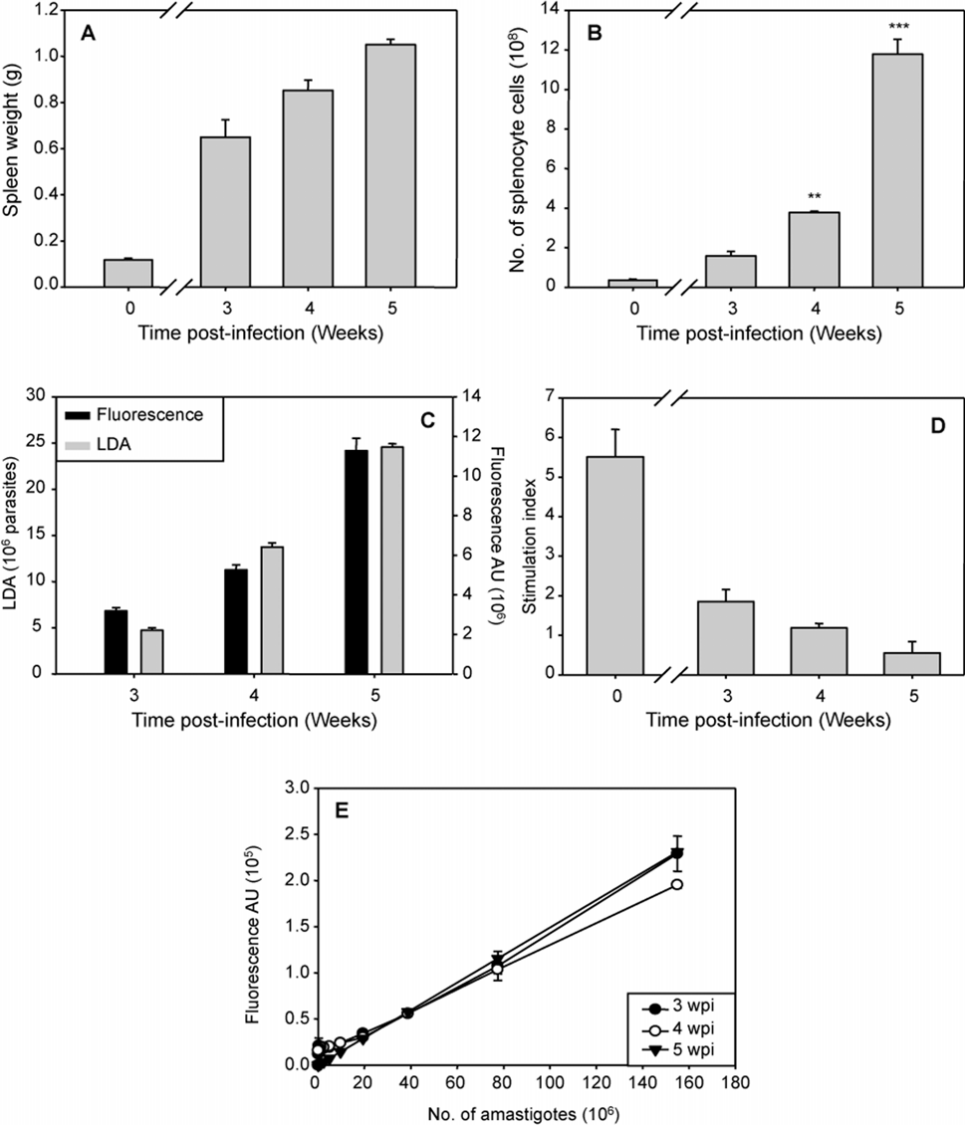
Establishing the appropriate time for *ex vivo* splenic explant culture. Mice infected with 10^7^ metacyclic-engineered HSP70+*L*. *infantum* strain were evaluated from 21 to 35 days post infection (n = 6 per time point). A) Spleen weight (shown as the mean ± SD). B) Splenocyte number obtained from a single animal was determined by counting the cells by direct microscopy (mean ± SD). C) Splenic parasite burden. Parasitic load expressed as LDAU after ten days of differentiation of amastigotes into promastigotes (left axis). The number of amastigotes (mean ± SD) was determined by fluorescent emission of splenocytes (10 mg of splenic cellular suspension) extrapolating to the standard curve (right axis) D) Splenocyte stimulation index expressed as the ratio ConA-stimulated and non-stimulated splenocytes (shown as the mean ± SD). E) Amastigote standard curve; correlation between fluorescent signal and number of amastigotes counted by microscopy. Significance level *** p< 0,001; ** p<0.01; from two tails of Student *t*-test.

Infection of laboratory mice with either *L*. *infantum* or *L*. *donovani* vary markedly between different organs. In the liver, the infection can resolve, unlike in the spleen, where *Leishmania* parasites may persist [32]. Therefore, we established a model of visceral *L*. *infantum* infection that allowed us to collect large numbers of naturally-infected cells from spleen to use as *ex vivo* explant. Infected mice were euthanized at different times post-infection. The spleens were weighed, processed to obtain a cellular suspension culture and the burden parasite and the proliferative response of mouse spleen cells were evaluated. There was a dramatic increase in spleen size, up to 1.0 g (Fig. 3A), splenocyte culture, (up to 12 x 10^8^ cell/spleen, Fig. 3B), and parasite burden (up to 25 x 10^6^/100 mg tissue, Fig. 3C), whereas the proliferative response dropped to basal levels at five wpi (Fig. 3D). Consequently, for drug screening we established five wpi as the time point for the *ex vivo* splenic explant culture. Finally, to be sure that fluorescence signal was stable in infected animals throughout all this time, parasites from infected spleens were also isolated and amastigote standard curves were prepared at 3, 4 and 5 wpi (Fig. 3E). As previously described by other authors, the loss of fluorescent signal during the evaluated period of time was not significant [18, 33]. In addition, the amastigote standard curves provided us the number of amastigotes that were used during the screening assays.

### Establishment and optimization of the *ex vivo* explant culture

**Fig 4:**
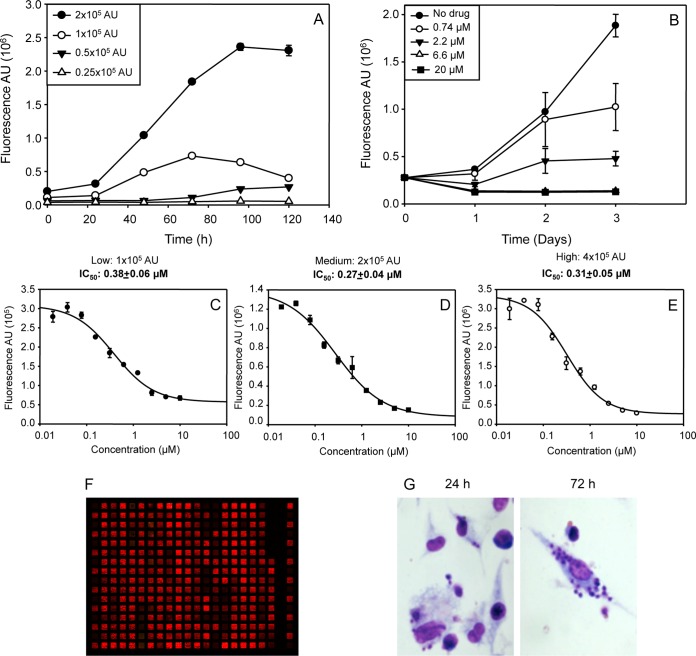
Establishing conditions for using the splenic explant culture as drug screening platform in 384-well plates. A) Starting cell density-well. Splenocytes were plated at different fluorescent signals (four 2-fold dilutions from 2.0 x 10^5^arbitrary units (AU) and incubated at 37ºC and 5% CO_2_ for 5 days. Each 24 h the fluorescent signal was read in an Odyssey® infrared imager (LI-COR). B) Effect of AmpB on splenocyte culture. Four 3-fold dilutions from 20 μM AmpB were added to splenic explant cultures at 2.0 x 10^5^ starting density-well. Fluorescence emission was recorded each 24 h during 3 days of incubation. C-E) AmpB dose-response profile. Splenocytes were plated at different starting densities and incubated in presence of ten 2-fold dilutions from 10 μM concentration. Fluorescence signal was recorded for 96 h. IC_50_ values were calculated after a nonlinear fitting with the SigmaPlot software. F) Infrared fluorescence recording of a representative 384-well plate in the infrared imager Odyssey (LI-COR). G) Giemsa staining of macrophages infected with HSP70+*L*. *infantum* 24 and 72 h after culturing. Experiments were carried out by triplicate and error bars represent standard deviations (SD).

Firstly, we checked the fluorescence dynamic range in which we could assure the effective and quantifiable replication of intracellular parasites inside macrophages in the absence of active drug over the course of the *ex vivo* culture. However, since there were individual-to-individual differences in parasite load, we first needed to establish the appropriate starting cell-density well that allowed the detection of parasite proliferation. For this purpose, we fixed the fluorescence level per well, instead of adjusting the number of cells. We followed the replication of the amastigotes within the splenic culture each 24 h during 5 days, using four different starting densities ranging from 0.25 x 10^5^ to 2.0 x 10^5^ arbitrary units (AU). The signal was measured in black 384-well plates with clear bottom using the infrared imager Odyssey (LI-COR). After 5 days of culture, only those wells containing 2.0 x 10^5^ AU at the beginning of the assay supported the active replication of the parasite in the absence of active drug over the whole experiment, indicating ongoing multiplication of the amastigote cells (Fig. 4A).

The next step was to assure that the selected starting cell density was accurate to guarantee the correct discrimination between drug-treated and untreated control wells. For doing this, different concentrations of AmpB (20, 6.6, 2.2, 0.74 μM) were added to the explant and the reduction in parasite load was measured by quantifying the loss of infrared fluorescence for 72 h (Fig. 4B). To confirm these results, and also to establish the AmpB_EC100_, as well as the appropriate period of incubation, ten serial 2-fold AmpB dilution steps covering a range from 10 to 0.02 μM were assayed against three starting cell densities (from 1.0, 2.0 and 4.0 x10^5^ AU). Readouts were harvested at 72 and 96 h. Surprisingly, IC_50_ values at 72 h were very similar in all the starting cell densities, ranging between 0.27–0.38 μM, (Fig. 4C-E) which corresponded with data collected by other authors [10, 34]. These results suggest that all these conditions allow the screening of compounds. To confirm the quality of the assay in discriminating active from inactive compounds, we calculated the Z prime (Z’) factor in 3 different screening experiments using 6 different plates. All Z’ were higher than 5.0, which demonstrated the correct reproducibility and the suitable quality of the assay [31].

Fig. 4F shows the fluorescence image of a 384-well plate containing the *ex vivo* culture treated with different compounds, where the red colour shows different intensities, which reflects the viability of the amastigotes in each well. The course of the *ex vivo* infection was followed over a period of 72 h by measuring the absolute fluorescence of the infection and the percentage of infected macrophages by classical Giemsa staining to determine parasite load (data not shown, Fig. 4G). No differences between both methods were accounted, thus pointing to the suitability of fluorescence analyses to assess the infectivity of HSP70+*L*. *infantum* strain on mouse macrophages.

**Fig 5:**
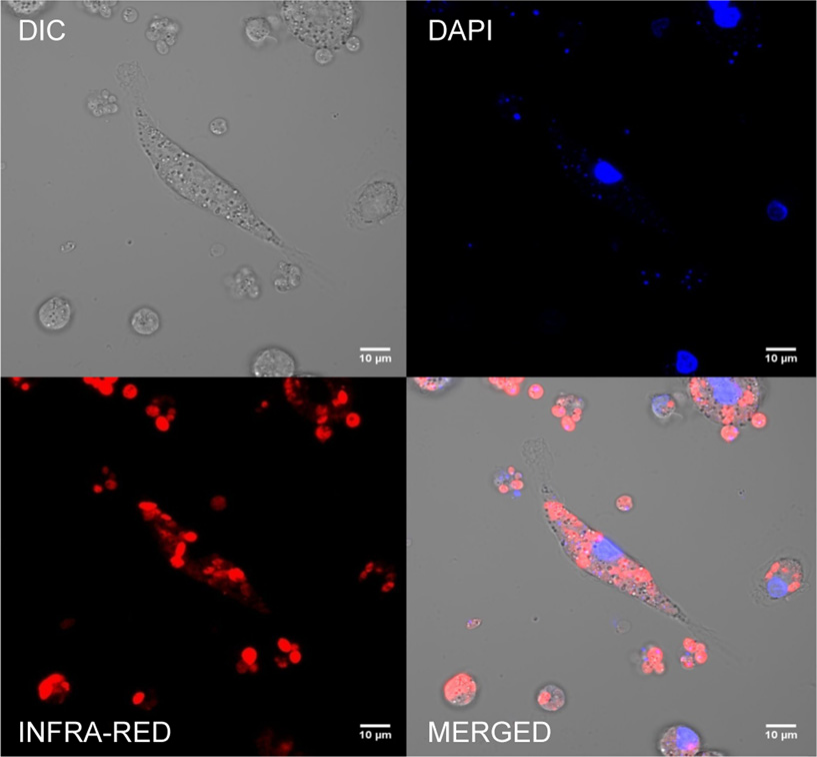
Confocal microscopy analysis of splenic explants infected with HSP70+*L*. *infantum* amastigotes. A) Differential Interference Contrast (DIC). B) HSP70+*L*. *infantum* emitting infrared fluorescence. C) DAPI staining of nucleic acids. D) Merged images. Microscopy images were acquired with a Zeiss LSM710 confocal microscope.

Moreover, intracellular forms were analyzed by confocal microscopy at 633 nm, after 5 wpi. A strong red fluorescence emission from round-shaped HSP70+*L*. *infantum*-emitting amastigotes was observed inside parasitophorous vacuoles in the cytoplasm of the infected macrophages (Fig. 5), confirming that the fluorescent signal was not lost during the ongoing proliferation of intracellular amastigotes.

### Screening of a 295-compound library

A library of 295 compounds comprising three collections of chemically distinct structures was screened at a concentration of 10 μM. The threshold for selecting hits was set as inhibitory activity of ≥ 60% after 3 independent assays. Twenty-four primary hits were obtained out of the 295 compounds (8%). Our next goal was to select, amongst the 24 confirmed hits, the best candidates for further investigation. Firstly, compounds were tested in dose-response curves using 7–8 concentrations 2-fold diluted from 50 μM, 100 mM and 2.5 mM respectively for combrestatins, carbolins and indenoisoquinolins. Then, the cytotoxicity of these compounds was tested in different concentrations with a 3-day assay on non-infected *ex vivo* explant cultures (CC_50_) using Alamar Blue (data not shown). Five out of the 24 hits were selected based on a threshold of Selective Index (SI = CC_50_/IC_50_), score of ≥30. They included combrestatins (8%; 2/24), carbolins (8%; 2/24) as well as indenoisoquinolines (4%; 1/24) (Table 2).

**Table 2:** IC_50_ and CC_50_ of selected hits.

Compound	IC_50_ (μM)	CC_50_(μM)	SI = CC_50_/IC_50_
Combrestatin 1	0,12 ± 0,0013	4,33 ± 0,49	33,7
Combrestatin 2	0,071 ± 0,0033	2,65 ± 0,21	37,3
Carbolin 1	1,44 ± 0.05	>200	138
Carbolin 2	5,24 ± 0.26	>200	38
Indenoisoquinoline	0,0113 ± 0,0019	0,33 ± 0,050	30

SI: Ratio of CC_50_ to IC_50_. Data are displayed as mean ± SD of three independent experiments.

### Appraisal of *L*. *infantum in vivo* infection by infrared fluorescence imaging

**Fig 6:**
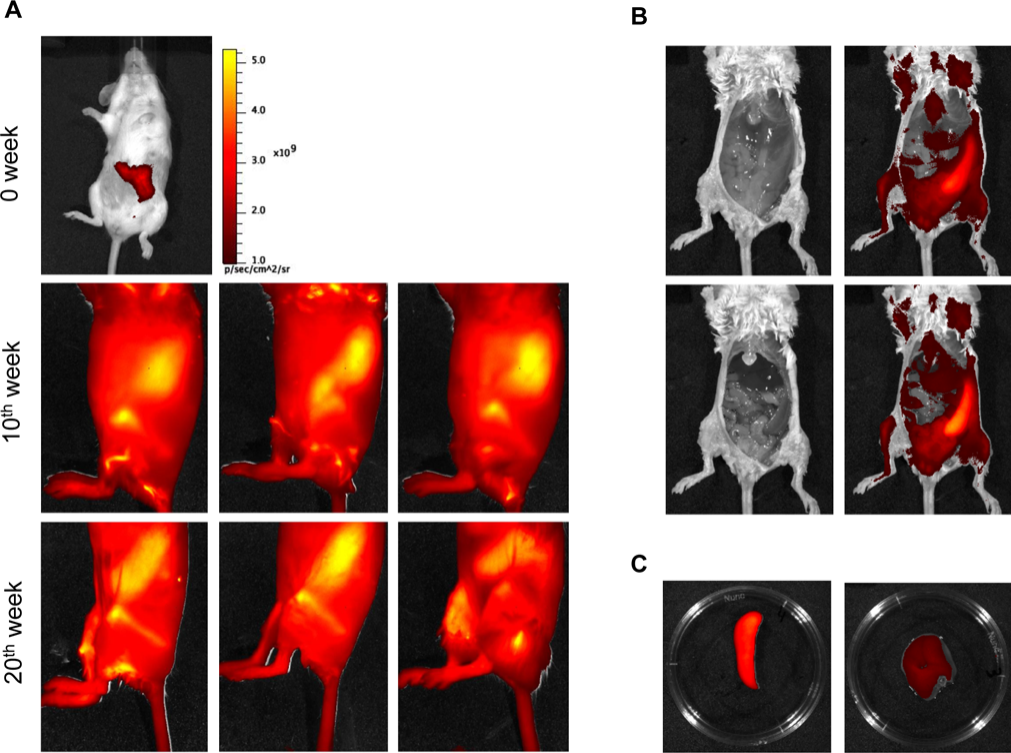
Monitoring the course of the in vivo visceral infection. A) *In vivo* imaging of BALB/c mice (n = 10) infected with 10^7^ metacyclic promastigotes of HSP70+*L*.*infantum* after 10 (three top) and 20 (three bottom) wpi. B) Mice (n = 5) were euthanatized and dissected, and the infrared signal was acquired after removal of skin and after removal of overlying peritoneum, showing internal organs after 10 wpi. C) Infrared fluorescence emission from a representative spleen (left) and liver (right) isolated from infected mice.

Finally, the applicability of the *HSP70*+*L*. *infantum* for imaging in BALB/C mouse was tested. Animals (n = 10) were inoculated intraperitoneally with 10^7^ stationary-phase HSP70+*L*. *infantum* promastigotes. The mice were imaged at 0, 10 and 20 wpi. A bright and intense signal was detected after parasite injection allowing even to trace the needle entry site. The transitory hepatic episode (14 days post-infection) was out of the scope of this experiment. On the contrary, spleen colonization was evidenced after 10 wpi and until the end of the experiment (Fig. 6A). Five animals were euthanized and after removal of the overlying skin (Fig. 6B top) and peritoneum (Fig. 6B down), increasing fluorescence signal was detected, confirming the above-mentioned results. The dissected organs were entirely fluorescent (Fig. 6C). Because of a lower parasite burden in the hepatic organ, the high level of fluorescence emitted by amastigotes in the spleen might have blocked the detection of parasites in the liver when imaging both organs at the same time. In order to tackle a future drug trial *in vivo*, the appraisal of infection was monitored for up to 2 wpi, showing that the fluorescent signal was still visible in the remaining group of mice (Fig. 6A). These results support future long-treatment schedules to tackle *in vivo* drug trials against VL.

## Discussion

In this work we present a potent tool based on infrared fluorescent proteins to achieve a double goal; on the one hand a robust platform for HTS screening of compounds and on the other hand an *in vivo* imaging of visceral *Leishmania* infections. Firstly, we chose between two different sources of infrared fluorescent proteins (*Deinococcus radiodurans* and *Rhodopseudomonas palustris*) [21, 22], and then we optimised the expression level by introducing different DSR of well-known virulence factors and heat shock proteins.

Initially, we compared the suitability of two fluorescent infrared stably-modified strains, in terms of brightness and fluorescence-emission properties in promastigotes and amastigotes of *L*. *infantum* BCN 150. iRFP+*L*.*infantum* reached significantly higher emission than IFP1.4+*L*.*infantum* throughout the different stages of *L*. *infatnum* life cycle. Fluorescent levels varied 2–3 times between both strains, and allowed the detection of as little as 100 intracellular amastigotes. In general, the intracellular stage has a lower emission due to their low metabolism, a fact that has been previously pointed out by other authors [35, 36]. Unlike other fluorescent reporters whose emission wavelengths are in the visible spectrum, near infrared fluorescent proteins have improved optical properties since the absorbance of haemoglobin, protein and lipids, as well as light-scattering, is minimal [21]. Thereafter, optimization of the vector was achieved by including different sequences; two of them previously described as virulence factors [23, 24], and the third one having a protection role against heat and oxidative stress in VL [25]. The highest levels of expression were associated with HSP70+*L*. *infantum*, which contains the downstream sequences from *HSP70-II* gene, with influence on transcript stability at conditions that facilitate the survival of the parasites within mammalian hosts [37]. Furthermore, the fluorescence emission of this reporter has been enhanced via stable integration into 18S rRNA locus, which represents an efficient and effective strategy that enables expression of proteins, avoiding the need of drug selection for both *in vitro* screenings and *in vivo* infections as previously described [17, 18, 35, 38, 39, 40, 41, 42].

Once the tool was selected, its applicability for drug discovery in VL in terms of searching new hits was assessed. *Leishmania* represents an enormous challenge as an intracellular parasite, since the drug must kill the parasite without affecting the host cells. Recently, HCS using image-based microscopy have been proposed as a robust platform for evaluating intracellular infections [10, 36]. In this system, axenically parasite cultures are used to infect macrophage’s cell lines. However, this approximation has several drawbacks: i) it is highly complex, ii) axenic parasites have notable differences with amastigotes [43], and when they have been used in screening assays a high false-positive rate was associated [36], and iii) the use of macrophage lines as host cell has also been questioned [34].

A novel approach based on the physiological and immunological environment of the infection site has been proposed [29]. *Ex vivo* explant is a primary culture that contains the full variety of cells that allow the parasite progression in the infected organ, and not only inside macrophages. Moreover, the infection is not recently established, but installed several weeks before the addition of the drug and the evaluation of its effect, and thus it greatly resembles human infections. From the lab point of view, the manipulation is minimal and a large number of test wells are easily prepared from a single infected animal.

In this work, the *ex vivo* splenic explant used for drug screening was developed in BALB/c mice infected with *L*. *infantum*, showing the typical features of VL with enlargement of spleens, high parasite burden, and loss of proliferation in splenic cells. The system congregates the basic rules previously defined for drug-screening systems for trypanosomatids [44]. i) it uses dividing populations of the mammalian stages of the parasite, ii) the drug activity is readily quantified by changes in infrared fluorescence signal, iii) a standard drug for VL (AmpB) showed activity at concentrations near to the one reached in serum [45, 46]. A crucial point was to establish the signal discrimination referred to the number of amastigotes in the infected cells per well.

The system was assessed with a selected set of compounds coming from three different collections and well-known active drugs were also included. The system adaptation presented here, allows for rapid identification of active compounds without the requirement of substrate reagents to get the readouts, and hence, the cost is reduced compared to other systems. The model uses a stable-modified *Leishmania* with no significant loss of reporter signal after more than 20 wpi. The fluorescence signal of the infected splenic explant has lineal correlation with the number of amastigotes, which makes the system easily adaptable to quantitative assays. Furthermore, there is no need of expensive infrastructure, such as the HCS, which are not affordable for some labs. Since the assay could be performed in 384-well plates, a single infected animal could be enough to screen more than 500 compounds.

However, host factors involved in the pharmacokinetics and pharmacodynamics of drugs are out of the scope of *in vitro* drug screening, consequently *in vivo* drug assays are still necessary. Using the same tool, parasite dissemination in an *in vivo* chronic model of VL has been traced for the first time based on infrared fluorescence signal. At 5 wpi, the animals were imaged, but not clear fluorescence signal was recorded. After 10 wpi an undisputed infrared signal was achieved, showing that the splenomegaly had been already established time before.

Many studies initiate drug administration trials after one or two weeks post-infection assessing the effect against the liver infection time with a peak in the parasite burden [47]. However, our goal was to use this model for longer times in order to perform future drug administrations against spleen infection, so the animals were pictured after 20 wpi and the fluorescence signal was still strongly detected. Therefore, this *in vivo* imaging model could be used without significant loss of reporter emission, allowing the appraisal of chronic infections after drug treatments.

In conclusion, the near-infrared fluorescent *L*. *infantum* strain used in the current work represents an important step forward in bioimaging research of VL, providing a robust model of phenotypic screening suitable for HTS of collections or libraries of small molecules in the amastigote form of mouse splenic explants. In addition, this novel *L*. *infantum* strain could be used in preclinical *in vivo* studies of potential lead compounds against visceralizing *Leishmania* species.
